# Latent profiles of job embeddedness and their predictors among specialist nurses in China

**DOI:** 10.3389/fpsyg.2025.1604014

**Published:** 2025-07-21

**Authors:** Yuting Fan, Jijun Wu, Yuxin Li, Xiaoli Zhong, Cui Chen, Jiquan Zhang, Lin He

**Affiliations:** ^1^School of Nursing, Chengdu University, Chengdu, China; ^2^Department of Nursing, Deyang People's Hospital, Deyang, China

**Keywords:** job embeddedness, professional mission, latent profile analysis, specialist nurses, influencing factors

## Abstract

**Objective:**

The aim of this study is to identify potential categories of job embeddedness among specialist nurses using latent profile analysis, explore the demographic characteristics of each subgroup, and examine the relationship between these profiles and their sense of professional mission.

**Methods:**

From March to April 2024, 492 specialist nurses from six general hospitals in Sichuan Province, China, were selected in this study using convenience sampling. A socio-demographic characteristic questionnaire, job embeddedness scale, and professional mission scale were used. Mplus 8.3 was used to explore the potential subgroups of job embeddedness among specialist nurses by latent profile analysis. IBM SPSS 26.0 was used to analyze the factors influencing the job embeddedness of specialist nurses in each category using univariate and Multinomial logistic regression analyses.

**Results:**

484 specialist nurses were finally included. Specialist nurses’ job embeddedness score was (27.63 ± 4.89). Specialist nurses’ job embeddedness could be categorized into three potential profiles: low embedded-alienation group (*n* = 113, 23.4%), medium job-embedded group (*n* = 199, 41.1%), and high embedded-identity group (*n* = 172, 35.5%). The results of the multivariate logistic regression showed that unmarried (OR = 0.087, *p* = 0.045), ≤3 years of specialty nursing experience (OR = 0.093, *p* = 0.029), choosing a nursing specialty based on personal interest (OR = 4.854, *p* = 0.013), and good and fair self-assessed health (OR = 10.211, *p* = 0.002 OR = 9.682, *p* = 0.002; OR = 10.656, *p* = 0.028; OR = 9.269, *p* = 0.037), low and moderate work intensity (OR = 5.719, *p* = 0.046; OR = 4.002, *p* = 0.017), and sense of professional mission (OR = 1.559, *p* < 0.001; OR = 2.542, *p* < 0.001) were the main factors influencing the job embeddedness potential profile of specialist nurses (all *p* < 0.05).

**Conclusion:**

There is significant heterogeneity in job embeddedness among specialist nurses, with medium job-embedded group and high embedded-identity groups dominating. Nursing managers should develop targeted intervention strategies based on the characteristics of different job embeddedness types of specialist nurses, such as optimizing human resource allocation and strengthening positive publicity for specialist nurses to stimulate a sense of professional mission among specialist nurses, which will enhance job embeddedness and reduce the turnover rate of specialist nurses.

## 1 Introduction

With the development of the social economy and the enhancement of public health awareness, the nursing profession is moving towards specialization. As the core members of the specialized nursing team, specialist nurses are defined internationally as registered nurses with a master’s degree or higher who have extensive clinical experience in a specific field, have received systematic training, and have obtained certified qualifications (Jokiniemi et al., 2021). In China, specialist nurses are defined as nursing personnel with college degree or higher who have completed the specialized nurse training organized by provincial or national nursing associations, passed the examination, and obtained the corresponding qualification certificate (Tian et al., 2024). They play an irreplaceable role in improving the quality of patient care, reducing medical costs, and promoting the development of nursing discipline (Horton et al., 2024). Specialist nurses originated in the United States and have gradually formed a specialized and standardized training and use system (Jokiniemi et al., 2021). The National Nursing Career Development Plan (2021–2025) emphasizes that high-quality, specialized nursing talents are the driving force for the development of the nursing industry and that China needs to strengthen the construction of a specialized nurse team and expand the supply of specialized nursing services in order to promote the system of specialized nursing services and improve the overall quality of nursing service capacity (National Health and Wellness Commission, 2022). However, compared with the more mature specialized nurse training system in foreign countries, China is still in the exploratory stage. Specialist nurses have relatively insufficient talent reserves, unsound management systems, and ill-defined job responsibilities, making it necessary to take on multiple roles, such as management, teaching, consulting, and scientific research, when undertaking clinical treatment work (Ding et al., 2021). Long-term high-intensity, multi-tasking work mode easily leads to role conflict and a sense of alienation, which affects their physical and mental health, reduces their organizational commitment and embeddedness in their work, and exacerbates staff turnover (Zhang et al., 2024; Çiçek Korkmaz and Torlak, 2024).

The concept of job embeddedness was first proposed by American psychologist Mitchell in 2001, aiming at explaining the reasons for employees’ active departure behavior from the perspective of job retention (Xue et al., 2023). Recently, it has become a hot research topic in management, sociology, and psychology. Job embeddedness refers to the network of close relationships between an individual and all work-related situations inside and outside the organization and can be used to measure the degree of connection, fit, and sacrifice between employees and the organization (Atalla et al., 2024). In this context, connection refers to the formal or informal links that nurses have with organizations, activities, and others; fit refers to nurses’ subjective perceptions of compatibility with the organization and the work environment; sacrifice refers to the personal loss that nurses may face if they leave an existing organization (Wang et al., 2024). Previous studies have shown that nurses’ job embeddedness is moderate and related to individual, organizational, and social factors such as psychological empowerment, years of experience, and organizational climate (Li et al., 2019). In addition, studies have shown that lower levels of job embeddedness may result in nurses’ deviation from their job roles, negative attitudes toward their jobs, decreased work engagement, and increased turnover (Jiang et al., 2012). On the contrary, a higher level of job embeddedness not only reduces nurses’ absenteeism but also helps to ensure patient safety and enhance team cohesion, promoting the construction of a quality nursing service system (Liu et al., 2022; El-Sayed et al., 2023). Therefore, an in-depth discussion of nurses’ current job embeddedness and analysis of its antecedent variables are crucial to optimizing nursing human resource management, improving the overall quality of nursing services, and achieving the goal of sustainable development.

Sense of professional mission refers to the individual’s sense of purpose and responsibility for their career, as well as the recognition of the value and significance of their career, which is mainly manifested in orientation, altruistic contribution, and proactive, enterprising (Xie et al., 2024). Research has shown that a sense of professional mission, as an important internal driving force for sustained engagement, helps mobilize an individual’s cognitive and emotional resources, enhances an individual’s professional resilience and organizational awareness, and enables them to demonstrate higher motivation and resilience to stress in the workplace, and reducing the loss of resources due to leaving the job (Cao et al., 2019). Job embeddedness theory states that the extent to which an individual stays in an organization depends on the connection, fit, and sacrifice between them and the organizational environment (Kiazad et al., 2015). Nurses with a high sense of professional mission often see their career aspirations as consistent with the organization’s direction, enhancing the fit between the individual and the work environment and gaining a sense of meaning and accomplishment at work (Liu et al., 2024). This positive experience may enhance nurses’ sense of identity and belonging to their work and strengthen their emotional connection with the organization, prompting them to become more embedded in their work at the emotional, cognitive, and behavioral levels. In addition, Kim and Park (2023) concluded that positive nursing professionalism significantly predicted job embeddedness. Nurses with higher levels of professionalism are more likely to develop a sense of identity, responsibility, and belonging to the organization, increase their fit, reduce their willingness to leave the organization and their actual departure behavior, and increase their career stability.

Current research on job embeddedness focuses on clinical nurses and explores its relationship with demographic and organizational characteristics, but there is a lack of research on the professional mission and job embeddedness of specialist nurses. As an important force in promoting the professionalization of nursing and meeting diversified health needs, specialist nurses’ job embeddedness not only affects their career development and willingness to practice but also has a bearing on the stability of the nursing workforce and the improvement of service quality. However, most previous studies have used variable-centered analysis methods, which makes it difficult to reveal the potential heterogeneity within the sample. Latent profile analysis (LPA) is an individual-centered statistical method that categorizes samples and identifies potential subgroups with similar characteristics by analyzing individual response patterns to each variable (Aflaki et al., 2022). Compared with traditional methods, LPA can visualize the distribution ratio of different categories of people and their characteristics. Therefore, this study used latent profile analysis to explore the different types of job embeddedness of specialist nurses and their influencing factors, aiming to provide a reference basis for nursing managers to develop targeted intervention strategies and improve the quality of nursing services.

## 2 Methods

### 2.1 Study design and participants

This study was a cross-sectional study. The convenience sampling method was used to select specialist nurses from six general hospitals in western China from March to April 2024 for the study. Inclusion criteria: (1) certified nurses and in service; (2) holding specialist nurse certification; (3) working in specialty nursing for ≥12 months; (4) informed consent and voluntary participation in this survey. Exclusion criteria: (1) nurses in training; (2) nurses who were not on duty during the study period due to sick leave, maternity leave, and personal leave.

### 2.2 Sample size

According to the sample size calculation method of Logistic regression analysis, the sample size is 10–15 times the number of independent variables (Ni et al., 2010). There are 20 variables in this study. Among them, 16 demographic variables, 1 dimension of job embeddedness, and 3 dimensions of professional mission. Considering the 20% sample attrition rate (questionnaires with illogical or regular answers), the sample size should be 250–375. In addition, potential profile analysis requires a sample size of at least 300 (Ferguson et al., 2020). A total of 492 questionnaires were collected in this study, and after excluding 8 questionnaires with illogical or regular responses, 484 samples were finally included for analysis.

### 2.3 Procedure

This study collected data through the online questionnaire platform “Questionnaire Star,” which has strict data encryption and privacy protection mechanisms. The study’s purpose, significance, and scale entries were imported into the e-platform, and a link/quadratic code was created for the questionnaire. Subsequently, the researcher contacted the relevant hospital directors, and after obtaining support and cooperation, a liaison officer was identified in each hospital, and training was provided to the liaison officers prior to the survey, covering the purpose of this study, how to fill in the questionnaire, and precautions to be taken. Furthermore, the link or QR code of the questionnaire was sent to the nursing workgroup by the liaison of each hospital. After obtaining full informed consent, respondents could access the questionnaire page through the link or scan the QR code to fill in the questionnaire. In order to ensure the validity and reliability of the data as much as possible, all the contents of the entries were set as mandatory. This study was set to be filled out anonymously on the computer or cell phone terminals, and each device was allowed to answer only once. All the questions could be submitted when they were completed. In order to guarantee the confidentiality of the research data and result, all questionnaire data were coded.

### 2.4 Measure

#### 2.4.1 Survey instruments

##### 2.4.1.1 Socio-demographic characteristic questionnaire

The socio-demographic characteristics questionnaire was self-developed by the researcher after reviewing the literature. It consists of two parts: socio-demographic characteristics of the specialist nurses, including gender, age, marital status, highest education level, self-assessed health status, and self-assessed sleep quality. Work-related information includes hospital level, department, title, position, mode of employment, years of working experience in specialist nursing, average monthly income, reasons for choosing the nursing specialty, self-assessed work intensity, and job satisfaction.

##### 2.4.1.2 Job embeddedness scale

The job embeddedness scale developed by Crossley et al. (2007) was used to assess the job embeddedness of specialist nurses. The scale consists of seven items in a single dimension and is based on a 5-point Likert scale ranging from 1 to 5, which is considered to be “strongly disagree” to “strongly agree,” with items 4 and 6 being reverse scored, and the total score ranging from 7 to 35. Higher scores indicate higher levels of job embeddedness among specialist nurses. The Cronbach’s alpha coefficient for this scale was 0.880. In this study, the Cronbach’s alpha coefficient was 0.874, which is good reliability.

##### 2.4.1.3 Professional mission scale

The scale was developed by Dobrow and Tosti-Kharas (2011) in 2011 and was Chineseized and revised by Zhang (2015) based on our cultural background. It has been widely used in the nursing community. It consists of three dimensions: orientation (4 items, e.g., “I feel destined to pursue my current career”), altruistic contribution (3 items, e.g., “My work contributes to society”), and proactive (3 items, e.g., “I am committed to my career”). The scale is scored on a 5-point scale ranging from “not at all” to “completely,” corresponding to a score of 1–5 respectively; the total score is 10–50. The higher the score, the higher the degree of professional mission. The Cronbach’s *α* coefficient of the scale is 0.840. In this study, the Cronbach’s α coefficient is 0.908.

#### 2.4.2 Statistical analysis

Mplus 8.3 was used to analyze the latent profile of job embeddedness in specialist nurses. The raw scores of the seven items from the job embeddedness scale were included as continuous observed variables in the model, starting with a single-category model, the number of latent categories was gradually increased until the model fit reached its optimal level. Model fit evaluation metrics are categorized into three types: ① Information metrics include the Akaike Information Criterion (AIC), Bayesian Information Criterion (BIC), and adjusted Bayesian Information Criterion (aBIC), with lower values indicating better model fit; ② Likelihood ratio test indicators include the corrected likelihood ratio test (LMR) and the bootstrap-based likelihood ratio test (BLRT). When *p* < 0.05, it indicates that the k-category model is superior to the k-1-category model. ③ Classification indicators include entropy (Entropy), which has values ranging from 0 to 1. The closer the value is to 1, the more precise the classification (Tein et al., 2013). Additionally, the optimal model generally has an average sample size of 50 per profile, and the smallest profile has at least 5 % of the total sample size (Wang et al., 2025)). After determining the optimal model, SPSS 26.0 was used for subsequent statistical analyses. Group differences across latent classes were tested using chi-square tests or Fisher’s exact tests (when expected cell counts were <5) for categorical variables, and one-way ANOVA for continuous variables. Furthermore, a multinomial logistic regression model was fitted using the latent profile membership as the dependent variable and the variables with statistical significance in the univariate analysis as independent variables. All categorical predictors were dummy-coded before inclusion in the model. For example, marital status was coded using “divorced/widowed” as the reference category. Sense of professional mission was treated as a continuous variable and entered using its original scale score. A *p*-value < 0.05 was considered statistically significant.

#### 2.4.3 Ethical considerations

The study strictly followed the ethical principles required by the Declaration of Helsinki and was approved by the People’s Hospital of Deyang City, Sichuan Province, China, under Grant No. 2024-04-016-K01. Before the survey, participants were informed of the purpose of the study and were assured of voluntary participation. All information was filled out anonymously and in complete confidentiality, and the study data were restricted to internal use by the research team only. Participants could withdraw at any time during the completion process.

## 3 Results

### 3.1 Latent profile analysis of specialist nurses’ job embeddedness

This study used the scores from the original project as an explicit indicator for specialist nurses’ job embeddedness. Starting from a single category, models ranging from 1 to 5 were sequentially fitted, with the fitting indices for each model shown in Table 1. The results indicate that as the number of categories increases, the AIC, BIC, and aBIC values gradually decrease. The entropy values for all five models exceed 0.8. However, when the number of categories was 5, although all fitting indices were within acceptable ranges, the minimum potential subgroup proportion was 3.7% (<5%), below the recommended minimum threshold, indicating insufficient model representativeness; when the model had four categories, the LMR value was not statistically significant (*p* > 0.05), indicating that model 4 was not significantly superior to model 3. In contrast, model 3 exhibits more ideal metrics, with an entropy value of 0.939 and average membership probabilities ranging from 95.8% to 98.7%, indicating high classification quality. Considering statistical fit and interpretability comprehensively, model 3 was ultimately selected as the optimal latent profile analysis model for the work integration of specialized nurses (Table 2).

**Table 1:** Fit indices of latent profile models for job embeddedness among specialist nurses.

Model.	AIC	BIC	aBIC	Entropy	*p*-value		Latent class probability
					LMR	BLRT	
1	9079.998	9138.547	9094.112	-	-	-	-
2	7776.586	7868.592	7798.766	0.858	0.017	<0.001	0.461/0.539
3	7164.323	7289.785	7194.567	0.939	0.001	<0.001	0.234/0.411/0.355
4	7001.272	7160.192	7039.582	0.947	0.115	<0.001	0.246/0.052/0.366/0.337
5	6166.706	6359.082	6213.081	0.988	0.036	<0.001	0.037/0.353/0.223/0.066/0.320

**Table 2:** Probability matrix for attribution of 3 potential profile types for specialist nurses’ job embeddedness.

Potential profile type	Probability of attribution to potential category (%)		
	C1	C2	C3
1	0.987	0.013	<0.001
2	0.022	0.958	0.021
3	<0.001	0.02	0.98

**Figure 1:**
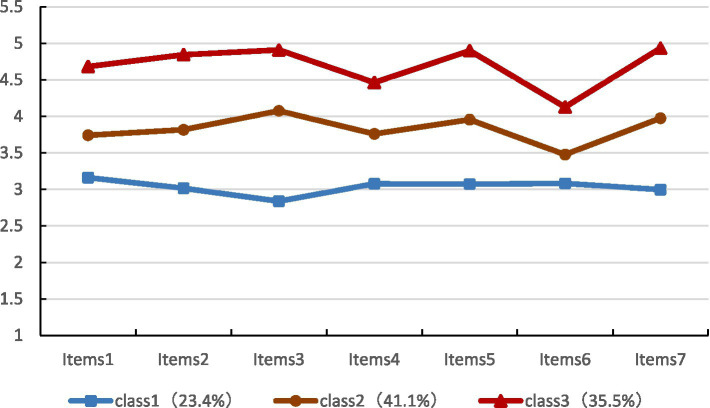
Latent profile overview of job embeddedness among specialist nurses.

The distribution of the three latent profiles across the seven job-embedded entries is shown in Figure 1, and the three categories of job-embedded nurse specialists were named according to the scoring characteristics of each entry. As shown in Figure 1, category 1, with a total of 113 (23.4%), had a generally low score for each item and the lowest score for “I like my current organization so much that I will not leave it,” so it was named the “low embedded-alienation group.” Category 2, with a total of 199 respondents (41.1%), has a medium level of scores between the other two groups, so it is named the “medium job-embedded group.” Category 3, with a total of 172 participants (35.5%), had significantly higher scores than category 1 and category 2 and scored the highest on the item “I feel connected to the organization,” so it was named the “high embedded-identity group.”

### 3.2 Univariate analysis of potential categories of specialist nurses’ job embeddedness

The details of the included respondents are shown in Table 3. The results of the univariate analysis showed that predicted class membership across the three latent classes of specialist nurses were statistically significant in terms of age, marital status, years of experience in specialty nursing, average monthly income, reasons for choosing the nursing profession, self-assessed health status, self-assessed sleep quality, self-assessed work intensity, self-assessed job satisfaction, and sense of professional mission (all *p* < 0.05).

**Table 3:** Univariate analysis of potential categories of specialist nurses’ job embeddedness.

Variables		C1 (*n* = 113)	C2 (*n* = 199)	C3 (*n* = 172)	χ^2^/*F*	*p*
Age	<30	33 (29.2%)	43 (21.6%)	22 (12.8%)	23.789	<0.001
	30–40	70 (61.9%)	133 (66.8%)	108 (62.8%)		
	>40	10 (8.8%)	23 (11.6%)	42 (24.4%)		
Marital status	Unmarried	47 (41.6%)	39 (19.6%)	10 (5.8%)	55.765	<0.001
	Married	60 (53.1%)	145 (72.9%)	151 (87.8%)		
	Divorced/widowed	6 (5.3%)	15 (7.5%)	11 (6.4%)		
Years of experience in specialized nursing	≤3	25 (22.1%)	7 (3.5%)	3 (1.7%)	82.449	<0.001
	4– <10	32 (28.3%)	70 (35.2%)	30 (17.4%)		
	10– <15	46 (40.7%)	76 (38.2%)	71 (41.3%)		
	≥15	10 (8.8%)	46 (23.1%)	68 (39.5%)		
Average monthly income	<5,000	45 (39.8%)	49 (24.6%)	28 (16.3%)	23.414	0.001
	5,000 ~ <8,000	49 (43.4%)	104 (52.3%)	95 (55.2%)		
	8,000 ~ <11,000	16 (14.2%)	29 (14.6%)	31 (18.0%)		
	≥11,000	3 (2.7%)	17 (8.5%)	18 (10.5%)		
The reason for choosing the nursing profession	Personal Interests	20 (17.7%)	94 (47.2%)	91 (52.9%)	45.046	<0.001
	Advice from others	68 (60.2%)	90 (45.2%)	68 (39.5%)		
	Specialized transfers	25 (22.1%)	15 (7.5%)	13 (7.6%)		
Self-assessed health status	Good	26 (23.0%)	102 (51.3%)	106 (61.6%)	104.060	<0.001
	Fair	48 (42.5%)	89 (44.7%)	62 (36.0%)		
	Poor	39 (34.5%)	8 (4.0%)	4 (2.3%)		
Self-assessed sleep quality	Good	24 (21.2%)	63 (31.7%)	92 (53.5%)	47.378	<0.001
	Fair	51 (45.1%)	103 (51.8%)	62 (36.0%)		
	Poor	38 (33.6%)	33 (16.6%)	18 (10.5%)		
Self-assessed work intensity	Low	14 (12.4%)	22 (11.1%)	29 (16.9%)	27.830	<0.001
	Middle	47 (41.6%)	126 (63.3%)	110 (64.0%)		
	High	52 (46.0%)	51 (25.6%)	33 (19.2%)		
Self-assessed job satisfaction	Satisfied	44 (38.9%)	118 (59.3%)	135 (78.5%)	48.811	<0.001
	Neutral	54 (47.8%)	71 (35.7%)	31 (18.0%)		
	Dissatisfied	15 (13.3%)	10 (5.0%)	6 (3.5%)		
Professional mission		31.15 ± 5.18	39.14 ± 3.36	44.30 ± 2.92	423.421	<0.001

### 3.3 Multivariate logistic regression analysis of specialist nurses’ job embeddedness

All variables were tested for multicollinearity, and the results showed that the tolerance of all explanatory variables was >0.1 and VIF < 5, indicating that multicollinearity was not a concern in the analysis. A multivariate logistic regression analysis was performed with the three categories of specialist nurses’ job embeddedness as dependent variables (“low embedded-alienation group” as the reference group) and the statistically significant variables of the univariate analysis as independent variables. The results showed that marital status (unmarried, married, divorced/widowed), years of work experience in specialty nursing (≤3 years, 4–<10 years, 10–<15 years, ≥15 years), reasons for choosing the nursing profession (personal interest, others’ advice, professional adjustment), self-assessed health status (good, fair, poor), self-assessed work intensity (low, moderate, high), and sense of professional mission (continuous variable) were the influencing factors of the predicted class membership according to the job embeddedness profile of specialist nurses (all *p* < 0.05), and the specific information is shown in Table 4. In this study, the “low embedded-alienation group” was used as the reference category. An odds ratio (OR) greater than 1 indicates a higher likelihood of belonging to a specific category relative to the reference category; an OR less than 1 indicates a lower likelihood of belonging to a specific category. As shown in Table 4, unmarried (OR = 0.087, *p* = 0.045) and those with ≤3 years of work experience in specialty nursing (OR = 0.093, *p* = 0.029) were more likely to belong to the low embedded-alienation group. Specialist nurses who chose nursing as a profession due to personal interest (OR = 4.854, *p* = 0.013), were more likely to belong to the medium job-embedded group. Further, specialist nurses who self-assessed work intensity as low (OR = 5.719, *p* = 0.046); or moderate (OR = 4.002, *p* = 0.017), were more likely to belong to the high embedded-identity group. Lastly, specialist nurses whose self-assessed health status was classified as good (OR = 10.211, *p* = 0.002) or fair (OR = 9.682, *p* = 0.002) and had higher scores for sense of professional mission (OR = 1.599, *p* < 0.001), were more likely to belong to the medium job-embedded group. Similarly, specialist nurses whose self-assessed health status was classified as good (OR = 10.656, *p* = 0.028) or fair (OR = 9.269, *p* = 0.037) and had higher scores for sense of professional mission (OR = 2.542, *p* < 0.001), were more likely to belong to the high embedded-identity group.

**Table 4:** Multifactorial analysis of potential categories of specialist nurses’ job embeddedness.

Variables		C2				C3			
		*β*	OR	95%CI	*p*	*β*	OR	95%CI	*p*
Marital status	Unmarried	−1.523	0.218	(0.030, 1.590)	0.133	−2.438	0.087	(0.008, 0.946)	0.045
	Married	−0.570	0.565	(0.091, 3.508)	0.540	−0.496	0.609	(0.076, 4.876)	0.640
Years of experience in specialty nursing	≤3	−2.370	0.093	(0.011, 0.790)	0.029	−2.856	0.057	(0.003, 1.088)	0.057
	4– <10	−0.934	0.393	(0.077, 2.016)	0.263	−1.128	0.324	(0.051, 2.073)	0.234
	10– <15	−1.352	0.259	(0.057, 1.184)	0.081	−1.368	0.255	(0.047, 1.366)	0.111
Self-perceived work intensity	Low	0.645	1.906	(0.454, 8.002)	0.378	1.744	5.719	(1.030, 31.749)	0.046
	Middle	0.787	2.196	(0.901, 5.350)	0.083	1.387	4.002	(1.276, 12.553)	0.017
Self-assessed health status	Good	2.323	10.211	(2.281, 45.709)	0.002	2.366	10.656	(1.290, 88.066)	0.028
	Fair	2.270	9.682	(2.224, 42.153)	0.002	2.227	9.269	(1.149, 74.760)	0.037
Reasons for choosing the nursing profession	Personal Interests	1.580	4.854	(1.391, 16.944)	0.013	1.435	4.199	(0.777, 22.691)	0.096
	Advice from others	0.473	1.605	(0.486, 5.297)	0.437	0.272	1.313	(0.250, 6.908)	0.748
Total score of professional mission		0.444	1.559	(1.375, 1.768)	<0.001	0.933	2.542	(2.154, 2.999)	<0.001

## 4 Discussion

The present study found that specialist nurses’ job embeddedness could be categorized into three potential subgroups: low embedded-alienation group (23.4%), medium job-embedded group (41.4%), and high embedded-identity group (35.5%). Of these, the main characteristics of the low embedded-alienation group were unmarried, ≤3 years of specialty nursing experience, and poor self-assessed health. Medium job-embedded group was dominated by 30– < 40 years of age, average self-assessed health, and average self-assessed sleep quality. High embedded-identity group were predominantly ≥40 years of age, ≥10 years of specialty nursing experience, and had good self-assessed health. In addition, this study showed that the total job embeddedness score of specialist nurses was (27.63 ± 4.89), suggesting a medium-high level, which is higher than the results of related studies by Egyptian scholars (Sorour et al., 2024). Analyzing the reasons, on the one hand, may be related to the difference in the survey population. The respondents of this study were specialist nurses. Compared to general nurses, specialist nurses are usually systematically trained to take on more complex tasks and decision-making responsibilities in specific areas and may be more likely to gain a sense of meaning and self-efficacy in the organization, leading to less willingness to leave and higher levels of job embeddedness. On the other hand, it may be influenced by different countries’ background characteristics, such as cultural values and level of economic development. In recent years, the Chinese government has increasingly emphasized the training and development of nurse specialists and has implemented relevant policies and structural reforms, which may potentially increase the level of job embeddedness of nurse specialists (Xu et al., 2024). Based on this, it is recommended that nursing administrators improve promotion pathways and staff retention policies, optimize the allocation of human resources, and rationally distribute work assignments to reduce the workload of specialist nurses and enhance their enthusiasm for work. However, it is worth noting that high embeddedness also has a “double-edged sword” effect (Reitz et al., 2010). Although high embeddedness can help reduce staff turnover, excessive embeddedness may lead to passive retention, decreased innovative behavior, and resistance to organizational change. Therefore, nursing managers should dynamically assess nurses’ job satisfaction and willingness to develop while promoting the embeddedness of specialist nurses in order to avoid the negative effects of over-embeddedness.

Unmarried specialist nurses were more likely to belong to the low embedded-alienation group, consistent with the findings of previous studies (Giosan, 2004). To analyze the reason, unmarried specialist nurses are mostly a young group with high expectations for personal growth and career development. When the organization fails to respond effectively to their psychological needs regarding promotion channels, working environment, and salary, it may trigger their concern for external opportunities, which weakens their commitment and emotional investment in the current organization and reduces the degree of job embeddedness (Wong et al., 2024). Additionally, unmarried specialist nurses’ life rhythm is not yet stable, and the uncertainty of their emotional needs and partner choices may prompt them to consider leaving their positions for family reasons. Therefore, nursing managers should pay attention to the career development needs and life-stage challenges of unmarried specialist nurses. They can help them balance their work and life needs by providing personalized organizational support, which improves job embeddedness and reduces the intention to leave.

This study found that specialty work experience ≤3 years was more likely to be in the low embedded-alienation group, similar to Song’s findings (Song et al., 2024). On the one hand, due to the relative lack of experience in specialized work, specialist nurses with low seniority are prone to negative emotions and barriers to adaptation in the face of unexpected critical situations or pressure from the assessment system, which may reduce their job satisfaction and affects their commitment and embeddedness (Xu et al., 2025; Zhang et al., 2024). On the other hand, nursing relies heavily on teamwork. The relatively limited years of experience and interaction with the team of low-seniority specialist nurses may lead to a lack of solid interpersonal networks and a sense of belonging to the organization, which may reduce their emotional engagement in their work and potentially affect job embeddedness (Wang et al., 2024). Therefore, nursing managers provide targeted skills training for nurses with little experience as specialists to help them improve their professional competence and ability to cope with stress in order to enhance their self-confidence and job satisfaction. At the same time, a positive work environment and organizational climate should be created to promote team integration.

Specialist nurses with low and medium work intensity were 1.744 and 4.002 times more likely to be classified in the high embedded-identity group than the low embedded-alienation group, respectively. It indicated that specialist nurses with low and medium work intensity were more likely to enter the high embedded-identity group. Work intensity is one of the important indicators of workload and work pressure. Due to specialty nursing’s complexity, specialist nurses often face high work demands and overloaded workloads (Kemp et al., 2022). High stress levels usually accompany high-intensity work. Studies have shown that occupational stress is associated with poor work performance and health outcomes (Kong et al., 2020). Prolonged exposure to high-intensity, high-stress work environments may trigger negative psychological and behavioral responses in nurse specialists, such as emotional exhaustion, cognitive fatigue, and sleep disorders, which not only reduce their job satisfaction and efficiency but also weaken their attention, decision-making ability, and judgment, and increase the risk of medical errors (Zhang et al., 2022; Shah et al., 2021; Mosadeghrad, 2013). The increase in medical errors will, in turn, exacerbate work pressure, further leading to emotional fatigue and burnout, it may weakening specialist nurses’ commitment to their work and sense of belonging to the organization, forming a vicious circle that affects their job embeddedness (Bagheri Hosseinabadi et al., 2019). Therefore, nursing managers pay attention to the work intensity of specialist nurses, rationally optimize the allocation of human resources, and reduce the workload. Additionally, establish an effective stress management mechanism, provide psychological support and emotional channeling when necessary, communicate regularly, and listen to their inner thoughts.

This study showed that specialist nurses in good or fair health were more likely to belong to the medium job-embedded group and the high embedded-identity group. Specialist nurses in better health may have greater physical tolerance, better adapt to high-intensity work rhythms, and positively cope with the high stress and demands of nursing care, thus maintaining high work motivation and job fit. On the contrary, specialists nurses with poorer health status may face physiological challenges such as fatigue, chronic pain, or gastrointestinal disorders, which not only affect their work performance but also increase presenteeism (Wu et al., 2024). Previous studies have shown that the more severe the presenteeism, the less job-embedded the nurses are (Fan et al., 2024). Therefore, it is suggested that nursing managers pay attention to the health status of specialist nurses, schedule shifts flexibly, and rationally adjust work hours and task allocation. In addition, specialist nurses should be encouraged to actively participate in health management, such as regular medical checkups and scientific exercises, to improve their physical quality, reduce presenteeism, and enhance the degree of job embeddedness.

Specialist nurses motivated to choose a career by personal interest were 4.85 times more likely to be in the medium job-embedded group than in the low embedded-alienation group. The reason may be that interest is an important intrinsic driver for individuals (Zeng et al., 2022). When an individual’s interest is aligned with their career, it leads to greater motivation to learn and enthusiasm for work. This interest-driven work attitude can motivate nurses to focus more on clinical nursing work, enhancing their sense of professional identity and career acquisition (Feng et al., 2024). Previous studies have shown that a higher sense of professional identity can not only effectively alleviate negative emotions in clinical work but also enhance the subjective initiative of nurses, promote their positive emotional experience of the nursing career, and further improve their work embedded (Zhang and Xiao, 2022). Therefore, nursing managers should focus on cultivating nurses’ love for the nursing profession, encouraging specialist nurses to choose the appropriate direction of specialty nursing based on their interests, making clear career plans, and providing opportunities for outbound learning to promote their professional growth. In addition, the important role of specialty nursing in patients’ health promotion and social well-being is emphasized to enhance specialist nurses’ professional pride and sense of value so that they can maintain a high level of dedication and commitment in their clinical work to enhance their job embeddedness.

This study showed that compared with the low embedded-alienation group, specialist nurses with higher scores of sense of professional mission were more likely to belong to the medium job-embedded group and high embedded-identity group, similar to the findings of several previous studies (Lee et al., 2024; Yang and Chen, 2020). Nurses’ sense of professional mission reflects individuals’ subjective cognitive and emotional experience of professional meaning, and its formation is a dynamic process of continuous development. Job embeddedness theory suggests that the degree of connection and fit between individuals and their work environment and the sacrifices they make jointly influence their willingness to stay and their occupational behavior (Kiazad et al., 2015). A sense of professional mission, as a highly autonomous and internalized intrinsic motivation, may enhance the level of job embeddedness of nurse specialists by increasing their sense of fit between them and organizational values and deepening their emotional engagement with their professional role (Zhong et al., 2025). In addition, specialist nurses often have key responsibilities in specialty disease management, health education, and clinical decision-making and can directly influence patients’ health outcomes and care experience. This direct feedback mechanism helps to enhance their sense of recognition of the meaning and social value of their work and strengthen their sense of professional honor, thus stimulating their work enthusiasm, improving their work autonomy, and enhancing their connection with their professional roles so the level of job embeddedness is higher (Lu et al., 2025). Therefore, nursing administrators should focus on cultivating the professional mission of specialist nurses by establishing educational and practical programs related to the professional mission, enhancing the positive promotion of specialized nursing work in daily life, as it helps the public recognize the meaning and value of specialized nursing, leading to a more favorable evaluation. This recognition allows specialist nurses to feel that their efforts are acknowledged and supported, which in turn stimulates their sense of professional mission and increases their willingness to remain focused on and embedded in their nursing roles. Furthermore, encouraging nurses to actively participate in departmental management and clinical decision-making while appropriately empowering them can enhance their work experience and job satisfaction, fostering greater work embeddedness.

## 5 Limitations

This study has some limitations. First, although potential profile membership was derived through model classification rather than direct self-reporting, the predictor variables used in the multinomial logistic regression analyses were based on self-reported data collected at a single point in time, which may introduce common method bias. Second, this study employed a cross-sectional research design, making it impossible to determine the causal relationship between occupational sense of professional mission and job embeddedness. Future studies are recommended to adopt longitudinal or interventional study designs to validate the association between the two further. Third, the study data were collected through online self-assessment questionnaires, which may be subject to individual subjective biases. Specifically, participants may be more inclined to conform to socially acceptable standards when answering questions related to job assignments or work intensity. Future studies are recommended to incorporate third-party assessments to enhance the objectivity and reliability of research findings. Additionally, the study sample was drawn from six tertiary general hospitals in Sichuan Province, China, limiting its geographical representativeness. Given differences in cultural backgrounds, healthcare systems, and educational levels across regions, caution is advised when generalizing the study findings. Finally, the study only examined the impact of sociodemographic factors and sense of professional mission on the potential job engagement characteristics of specialist nurses, without conducting a comprehensive analysis from multiple perspectives such as personal resources and social resources. It is recommended that future studies further conduct multi-center, large-sample studies, expand the range of independent variables, and enrich statistical analysis methods to validate and refine the conclusions of this study.

## 6 Conclusion

This study used latent profile analysis to categorize the job embeddedness of specialist nurses in six general hospitals in Sichuan Province, China, and the results were classified into three categories: low embedded-alienation group (23.4%), medium job-embedded group (41.1%), and high embedded-identity group (35.5%). Multinomial logistic regression analysis showed that marital status, years of experience in specialty nursing, reasons for choosing the nursing specialty, self-assessed health status, self-perceived work intensity, and sense of professional mission were the key factors influencing the job embeddedness of specialist nurses. Based on this, nursing managers should pay attention to the job embeddedness level of specialist nurses and develop individualized and differentiated management strategies for different categories of specialist nurses to enhance their sense of professional mission and improve their job embeddedness.

### 6.1 Implications for nursing and health policy

According to the results of this study, in order to improve the level of job-embeddedness of specialist nurses, it is recommended that hospital administrators develop targeted intervention strategies. Helping specialist nurses recognize their role position and work value more clearly and develop a sense of professional mission by strengthening career development planning et al., thus enhancing their sense of job embeddedness. It could help optimize the construction of the specialist nurse workforce, promote the sustainable development of specialty nursing, and promote realizing the strategic goal of “Healthy China 2030.”

## Data Availability

The raw data supporting the conclusions of this article will be made available by the authors, without undue reservation.
